# Research on road surface damage detection based on SEA-YOLO v8

**DOI:** 10.1371/journal.pone.0324439

**Published:** 2025-06-18

**Authors:** Yuxi Zhao, Baoyong Shi, Xiaoguang Duan, Wenxing Zhu, Liying Ren, Chang Liao

**Affiliations:** 1 Jinan Zhuolun Intelligent Transportation Technology Co., LTD, Jinan, Shandong, China; 2 Liaocheng Inspection and Testing Center, Liaocheng, Shandong, China; 3 Linyi City Inspection and Testing Center, Linyi, Shandong, China; 4 Shandong University, Jinan, Shandong, China; 5 Pizhou City Public Security Bureau Traffic Police Brigade, Jiangsu, China; Kafkas University: Kafkas Universitesi, TÜRKIYE

## Abstract

Road damage detection is of great significance to traffic safety and road maintenance. However, the existing target detection technology still has shortcomings in accuracy, real-time and adaptability. In order to meet this challenge, this study constructed SEA-YOLO v8 model for road damage detection. Firstly, the SBS module is constructed to optimize the computational complexity, achieve real-time target detection under limited hardware resources, successfully reduce the model parameters, and make the model more lightweight; Secondly, we integrate the EMA attention mechanism module into the neck component, enabling the model to utilize feature information from different layers, enabling the model to selectively focus on key areas and improve feature representation; Then, an adaptive attention feature pyramid structure is proposed to enhance the feature fusion capability of the network; Finally, lightweight shared convolutional detection head (LSCD-Head) is introduced to improve feature representation and reduce the number of parameters. The experimental results on the RDD2022 dataset show that the SEA-YOLO v8 model has achieved 63.2% mAP50. The performance is better than yolov8 model and mainstream target detection model. This shows that in complex urban traffic scenarios, the model has high detection accuracy and adaptability, can accurately locate and detect road damage, save manpower and material resources, provide guidance for road damage assessment and maintenance, and promote the sustainable development of urban roads.

## 1. Introduction

### 1.1. Research background

Today, every country has a dense transportation network, which is extremely important for every country’s economy, society and national defense. Efficient transportation can reduce the cost of freight transportation time and significantly improve logistics efficiency. Roads constitute the fundamental artery of transportation systems, serving as a pivotal component of urban and rural infrastructure [1]. The integrity and optimal maintenance of road infrastructure are prerequisites for successful urbanization and economic growth. However, as the road network expands, the issue of road damage has become increasingly pressing [2,3]. The varying loads of vehicles and the natural lifespan of roads contribute to varying degrees of damage. Road defects, mainly including potholes and cracks, not only hinder the efficiency of road traffic, but also increase the instability of vehicle movement, increase the risk of traffic accidents, and pose a major threat to the safety of vehicles and passengers [4]. Currently, over 80 nations and regions globally boast a combined operational highway length surpassing 230,000 kilometers [5]. With the construction of highways in various countries, more and more roads begin to appear pavement damage, such as pavement cracking, which is aggravated by water seepage, which will rapidly reduce road quality and bring danger to driving vehicles, and road damage such as cracks and potholes will affect driving comfort and lead to traffic accidents. Without prompt identification and repair of road damages, these poor conditions can significantly increase vehicle wear and the risk of traffic incidents, contributing to further economic burdens. Statistics show that subpar road conditions contribute to 16% of all traffic accidents [6,7]. It is reported that traffic accidents caused by road potholes and crack collapse are on the rise. There were 4,775 and 3,564 reported accidents due to potholes in 2019 and 2020, respectively. Potholes alone are responsible for 2,600 deaths a year between 2016 and 2020, with potholes identified as the cause of 1% of 60,000 accidents on the road. Therefore, pavement damage detection is very important. The damage of road surface will not only cause the reduction of traffic efficiency, but also cause immeasurable impact on vehicles and pedestrians [8,9]. Therefore, pavement damage detection is very important. The damage of road surface will not only cause the reduction of traffic efficiency, but also cause immeasurable impact on vehicles and pedestrians.

Presently, methods for identifying road damage are categorized into three types: manual inspection, automated inspection, and image processing. In less developed countries, pavement assessment often depends on manual inspection, which is characterized by safety concerns, inefficiency, high expenses, and variability in judgment due to the reliance on individual inspectors’ expertise. Advances in technology have led to a growing trend toward automated road inspection, including the deployment of vehicles fitted with infrared or sensor technologies [10]. However, the intricate nature of road environments poses challenges for automated systems in achieving the necessary precision and speed for practical applications, while also imposing significant hardware and operational costs.

Image processing stands out for its efficiency and cost-effectiveness, with its accuracy continually improving alongside technological advancements.Consequently, numerous researchers have turned to image processing to identify pavement defects. Conventional image processing approaches typically involve the manual selection of features—such as color, texture, and shape—to isolate defects, followed by the application of machine learning algorithms for classification and matching purposes. Nevertheless, the complex road environment limits the ability of traditional image processing to meet the demands of real-world engineering in terms of model generalization and robustness when relying on hand-crafted feature extraction [11].

In light of these considerations, it is imperative to promptly detect and repair road damages in order to maintain the integrity of road infrastructure and enhance road safety. This task poses stringent demands on the field of road damage detection, necessitating advanced techniques and methodologies to ensure accurate and timely identification of road defects. In recent years, with the rapid development of deep neural networks, deep learning has received extensive attention in many fields and in the field of object detection. Deep neural networks provide superior speed and accuracy in target detection tasks, demonstrating strong robustness and generalization capabilities. By avoiding manual feature extraction and complex feature segmentation operations, deep learning minimizes the risk of misclassification or missing key target features during feature pre-sampling. Li et al. [12]. Proposed an improved Faster R-CNN road crack recognition model. Firstly, residual network ResNet50 was used as the backbone network for feature extraction in the Faster R-CNN model, and it was integrated with the squeezing and excitation network (SENet) to enhance the attention mechanism of the model. Sun et al. [13] proposed an improved algorithm based on YOLOv8. First, traditional convolution is replaced in the network backbone with a module consisting of spatial-to-depth layers and nonstrided convolution layers (SPD-Conv), which enhances the ability to identify defects of small size. The neck of YOLOv8 was then replaced with the neck of the ASF-YOLO network to fully integrate spatial and proportional features and improve multi-scale feature extraction capabilities. Finally, Wise-IOU (WIOU) is used to optimize the loss function of the model. Wan et al. [5]. Proposes a lightweight model for road damage identification by improving the YOLOv5s method. First, a new backbone network Shuffle-ECANet is proposed by adding an ECA focus module in the lightweight ShuffleNetV2 model. Secondly, in order to ensure the reliability of detection, we adopted BiFPN, which improved the capacity description characteristics of the network compared with the original feature pyramid network. Moreover, in the model training phase, localization loss is modified to Focal-EIOU in order to get higher-quality anchor box. Wang et al. [14]. Proposed a tracking model based on transformer optimization, Road-Transtrack. First, a YOLOV5-based classification network was used to divide the collected road damage images into two categories, namely potholes and cracks, and make a road injury dataset. Then, the proposed tracking model is improved by using transformer and self-attention mechanism.

### 1.2. Research objectives

Despite the remarkable achievements of current deep learning-based object detection technologies, there are still significant challenges and opportunities for improvement in the field of road injury detection. First, road injuries show a variety of irregular shapes and sizes, which makes the detection task very challenging [15,16]. Moreover, the current model still has the disadvantage of a large amount of computation, which makes it difficult to achieve real-time target detection with limited hardware resources [17]. Aiming at the above two deficiencies, the YOLOv8 model was improved in this study. The study made four contributions:

(1) The SBS module is constructed to optimize the computational complexity, achieve real-time target detection under limited hardware resources, successfully reduce the model parameters, and make the model more lightweight;
(2) Incorporating the EMA attention mechanism module into the neck component enables the model to utilize feature information from different layers, allowing the model to selectively focus on key areas and focus on important features of road defects, improving feature representation;
(3) Aiming at the defects with irregular shape and size in road detection, an adaptive feature pyramid structure is proposed, which enables the network to focus on features adaptively according to the size of defects, and enhances the ability of the model to detect road defects;
(4) A lightweight shared convolutional detection head (LSCD-Head) is introduced to improve feature representation and further reduce the number of parameters.

## 2. Related work

### 2.1. Road damage detection

The traditional approach to road damage detection, predominantly relying on vehicle patrols, manual photography, and inspectors, is not only laborious and inefficient but also demands considerable human resources [18]. Moreover, the need for patrol vehicles and personnel to occupy the road during inspections often leads to potential traffic congestion and safety concerns. Additionally, manual inspection heavily relies on the expertise and subjectivity of inspectors, introducing a degree of variability and uncertainty. However, with the remarkable advancements in computer vision, image segmentation techniques based on artificial feature extraction have emerged as a potential alternative [19]. These techniques utilize methods such as edge detection, directional gradient histograms, and wavelet transforms to identify road damage. Nonetheless, these approaches are limited by the necessity for manual feature extraction algorithm design, their inability to fully capture semantic information, and their subpar performance in complex scenarios [20]. Fortunately, deep learning-based object detection algorithms have offered a novel and promising solution for road damage detection [21]. These algorithms are capable of automatically pinpointing the precise location, type, and boundary information of damage within images, thereby enabling swift and accurate road damage detection. The current landscape sees numerous deep learning-driven object detection methods being successfully deployed for road detection tasks, heralding a new era in road maintenance and safety [22–24]. Long et al. [25] proposed RDD-YOLO, a road disease detection algorithm based on YOLOv7-tiny. Firstly, K-Means ++ algorithm is used to get the anchor frame that fits the target size better. Secondly, the Quantitative Sensing Reparameterization module (QARepVGG) is used in the small target detection branch to enhance shallow feature extraction, and three inputs embedded in the neck of the enhanced attention module are constructed to suppress complex background interference. In addition, lightweight decoupling head (S-DeHead) is constructed to improve the accuracy of small target detection. Finally, normalized Wasserstein distance measurement (NWD) is used to optimize the small target localization process and alleviate the problem of sample imbalance. This improved strategy makes up for the YOLO v7 model’s ability to pay attention to road defects under the interference of complex background, and improves the model’s detection ability.Nitin Nagesh et al. [26] proposed an automated method that can improve the detection accuracy of underground road voids by infrared (IR) images collected by UAVs. First, the framework relies on principal component thermal imaging analysis to improve the accuracy of damage detection, and then automates the detection process using the EfficientDet algorithm. EfficientDet is based on the EfficientNet backbone network. A composite scaling method is introduced to simultaneously adjust the depth, width and resolution of the network to achieve a balance between optimal performance and efficiency.

### 2.2. YOLO algorithm

**Fig 1:**
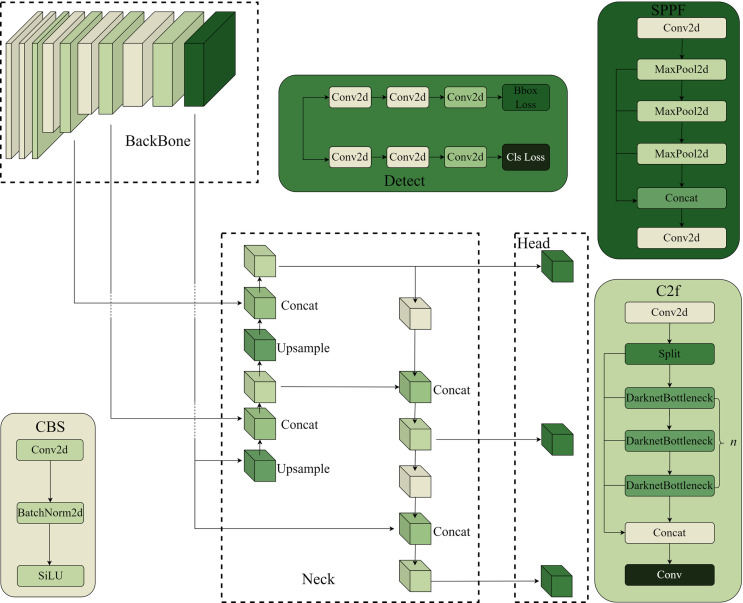
YOLO v8 Structure Diagram.

The YOLO (You Only Look Once) algorithm revolutionizes the field of object detection by reframing it as a regression problem. It predicts the precise location and classification of objects within an image grid using a single convolutional neural network, thereby eliminating the need for traditional candidate box extraction and enabling real-time object detection. YOLO v8, the latest iteration of this groundbreaking approach, builds upon the successes of previous YOLO generations by significantly enhancing its operational speed, reducing model parameters, and refining recognition accuracy [27–29]. The network architecture of YOLO v8, as depicted in Fig 1, demonstrates its ability to swiftly and precisely detect objects in complex environments. By optimizing its design, YOLO v8 continues to push the boundaries of object detection, offering a highly efficient and accurate solution for a wide range of applications.

The input component holds a pivotal role in preparing the input images for the neural network. Its tasks include resizing the images to a specified dimension, normalizing them to enhance data consistency, and converting them into a tensor format that is compatible with the network’s architecture. Furthermore, this component incorporates data augmentation techniques to enrich the training data. One such data augmentation technique is the Mosaic method, which ingeniously merges several training images into a single, enlarged image. This approach enables the model to process multiple images in parallel during a single forward pass, significantly enhancing training efficiency. The backbone component of the network is comprised of several key modules. These include CBS (Convolution, Batch Normalization, and Activation Function) blocks, C2f modules, and the Spatial Pyramid Pooling with Features (SPPF) module. Together, these modules transform the raw input image into a multi-scale, high-dimensional feature representation, ultimately forming a multi-scale feature map. Notably, during the feature extraction process, the output features are directly fed into the upsampling operation, eliminating the need for a 1 × 1 convolution before upsampling, as seen in previous iterations of the YOLO algorithm. This refinement not only reduces computational complexity but also preserves crucial spatial information, leading to improved detection performance. The C2f module, an evolution from the previous C3 module, retains its core design principles while introducing several advancements. Like the C3 module, it leverages CSPNet and the residual concept, dividing the network into two halves, each comprising multiple residual blocks. However, the C2f module stands out by incorporating more residual connections and introducing a unique splitting operation. This innovation eliminates convolutional operations within the branches, enriching the gradient flow during backpropagation and thus enriching the feature information. This strategic division not only decreases the model’s complexity but also enhances the efficiency of feature extraction. The SPPF module represents a significant shift from the traditional parallel approach, combining both parallel and serial processing [30–32]. This hybrid approach achieves a harmonious fusion of local and global information within the feature map. By preserving spatial information and adapting to a multi-scale pooling layer that caters to varying input sizes, the SPPF module ensures the integrity of the feature representation. Furthermore, the model utilizes the SiLU activation function, a smooth, nonlinear, and asymptotic alternative. In comparison to traditional Sigmoid and Tanh activation functions, SiLU offers superior performance, while circumventing the potential gradient explosion issues associated with the ReLU activation function. The mathematical formulation of the SiLU activation function is as follows:


$$SiLU\left(x\right)=x\times sigmoid\left(x\right)=\frac{x}{1+e^{-x}}$$ (1)

The Neck layer of the proposed network incorporates a diverse set of modules, including CBS, C2f, Upsample, and Concat, arranged in an FPN + PAN structure. This innovative configuration enables the network to seamlessly fuse feature information across multiple scales while maintaining spatial details. This approach is particularly advantageous in handling objects of varying sizes, thereby enhancing both object localization and classification accuracy. In the detection component, we adopt an anchorless detection method, which abandons the traditional anchor-based approach. Instead, it directly predicts the center point of the target and measures the distances from that center to the four edges of the object. This approach offers a more intuitive and efficient way of detecting objects. Moreover, we introduce a novel matching strategy, inspired by the Task-Aligned Assigner from the TOOD model. This strategy goes beyond the traditional IoU matching algorithm, employing a high-order combination of classification scores and IoU to assess the degree of task alignment. By doing so, it dynamically prioritizes high-quality anchors, further refining the detection performance. The mathematical formula that underpins this matching strategy is as follows:


$$t=s^{\alpha }\times u^{\beta }$$ (2)

The Neck layer of our proposed network comprises modules such as CBS, C2f, Upsample, and Concat, organized in an FPN + PAN structure. This architecture facilitates the integration of feature information across various scales while preserving spatial details, crucial for handling objects of diverse sizes and improving both localization and classification accuracy. In the detection phase, we adopt an anchorless approach that directly predicts the center point of the target and measures the distances to its four edges. Moreover, we introduce a matching strategy that utilizes a combination of the classification score(s) and the CIoU value(u) between the predicted frame and the ground truth frame. This Task-Aligned Assigner strategy, with weight hyperparameters α and β, allows for fine-tuning the degree of match. The product of s and u serves as a measure of alignment, where a t value close to 1 indicates a high degree of correspondence between the prediction and the ground truth, qualifying it as a positive sample. Utilizing t, we rank and select the top K prediction frames as positive samples, enhancing the model’s accuracy. In cases where a prediction frame aligns with multiple ground truth frames, we retain the one with the highest degree of match. This approach disentangles the prediction of target location and category, augmenting the model’s flexibility and adaptability to diverse detection tasks and scenarios. The loss function of our model comprises two components: classification and regression. For classification, we employ Binary Cross Entropy(BCE) to compute the loss, while for regression, we utilize Distributed Focus Loss(DFL) and CIoU loss. The BCE loss is calculated using the following formula:


$$L_{BCE}=\frac{1}{N}\sum_{i=1}^{N}L_{i}=\frac{1}{N}\sum_{i=1}^{N}-\left[y_{i}\log(p_{i})+(1-y_{i})\log(1-p_{i})\right]$$ (3)

In this context, N stands for the batch size, yi denotes the label value, and pi signifies the predicted value of the model. The formula for DFL is as follows:


$$S_{i}=\frac{y_{i+1}-y}{y_{i+1}-y_{i}}$$ (4)

$$DFL(S_{i},S_{i+1})=-((y_{i+1}-y)\log(S_{i})+(y-y_{i})\log(S_{i+1}))$$ (5)

Here, *y* represents the label value, and *y*_*i*_ and *y*_*i+1*_ are the two closest label values to *y*. The formula for CIoU is as follows:


$$L_{CIoU}=1-IoU+\left(\frac{\rho^{2}\left(b,b^{\delta t}\right)}{c^{2}}\right)+\alpha v$$ (6)

In these formulas, *b* and *b*^*δt*^ denote the center points of the predicted box and the ground truth box, *ρ* represents the Euclidean distance between these two center points, *c* represents the diagonal distance of the minimum enclosing box that can contain both the predicted box and the ground truth box simultaneously, *α* is a weight function, and *v* is used to measure aspect ratio consistency. During actual training, the model calculates total loss by weighting these three losses using a certain weight ratio.

## 3. The proposed method

**Fig 2:**
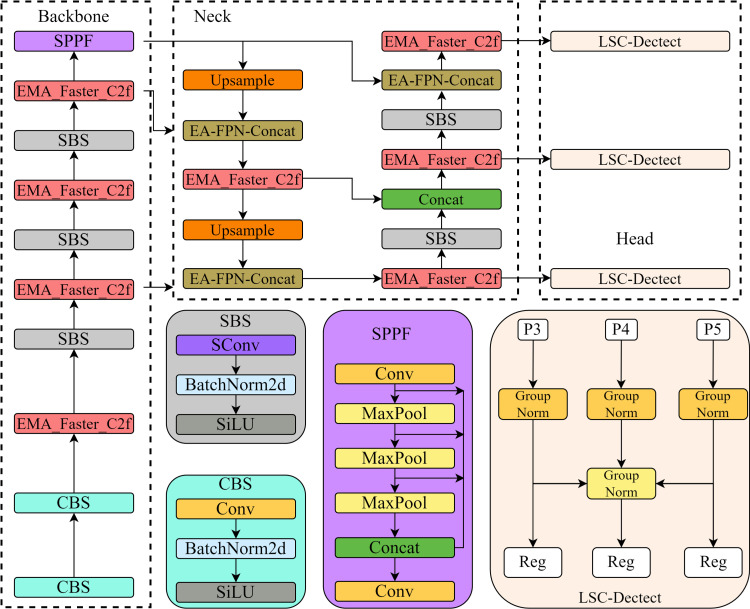
Improved YOLO v8.

In this article, YOLO v8 is improved as shown in Fig 2. The SBS module is added to the backbone network and Neck, which reduces the number of parameters and computation of the model, while maintaining the superior recognition performance of the model, and improves the performance and output of the model; the EMA_Faster_C2f module is constructed to further reduce the amount of redundant computation and the number of parameters and to improve the recognition performance of the model focusing on the important defect features; and by constructing the AE-FPN structure to enhance the model’s ability to detect road defects at different scales.

### 3.1. SBS module

**Fig 3:**
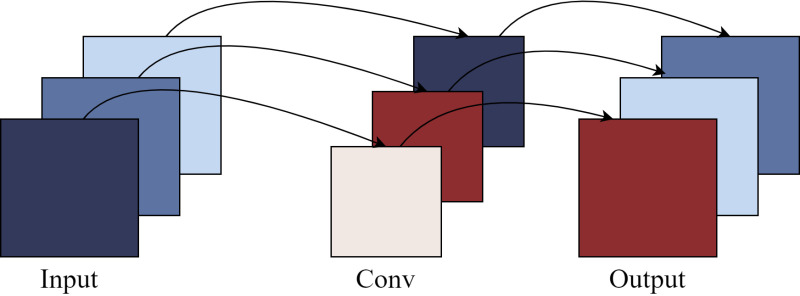
SBS Schematic Diagram.

The core premise of SConv revolves around the thorough disentanglement of channel correlation and spatial correlation within the feature mapping, thereby enabling the independent processing of these two dimensions. In contrast to traditional convolution methods, which concurrently learn both spatial and channel information in the feature layer during the training process, SConv facilitates a complete separation between channel mapping and spatial mapping. This specific operational approach is depicted in Fig 3, showcasing the distinctiveness and effectiveness of the proposed method.

By completely separating the channel correlation from the spatial correlation of the feature layer, SConv is able to extract high-level abstract features more efficiently compared to ordinary convolution. Ordinary convolution uses a convolution kernel of size $F\times F\times C\times C^{\prime }$ to convolve features of W×H×C to $W^{\prime }\times H^{\prime }\times C^{\prime }$. Compared to normal convolution, SConv possesses higher efficiency and can drastically reduce the number of parameters of the network, the number of parameters required for SConv is F×F×C, and the number of parameters required for the ordinary convolutional part is $F\times F\times C\times C^{\prime }$, It follows that the number of parameters required for SConv is 1/1C′\nulldelimiterspaceC′ of the number of ordinary convolutional parameters.

### 3.2. EMA_Faster_C2f module

In the realm of deep learning, the attention mechanism serves as an ingenious approach that emulates the human visual and cognitive systems. This mechanism significantly aids recommendation models by assigning varying degrees of importance to different parts of the input data. By doing so, it enables the extraction of more crucial and pertinent information, ultimately enhancing the model’s ability to make precise assessments. Remarkably, this approach does not impose additional computational or storage overhead, thus bolstering the overall performance and generalization capabilities of the model. In the context of road damage detection, the traditional YOLOv8n model’s straightforward feature fusion strategy encounters difficulties when confronted with the coexistence of large- and small-scale targets. Specifically, this approach tends to constrain the depth and richness of feature representation. Although the importance of the attention mechanism in augmenting feature representation is widely acknowledged, traditional channel dimensionality reduction methods can inadvertently compromise the integrity of deep visual information. To address this challenge, the EMA attention mechanism offers a unique solution. By avoiding dimensionality reduction, this mechanism achieves comprehensive information retention and computational efficiency through the reconstruction of a subset of channels and the uniform distribution of spatial semantics among sub-features. Not only does it globally encode information to modulate channel weights, but it also captures pixel-level relationships through cross-dimensional interactions. In the present study, the EMA module is seamlessly integrated into the EMA_Faster_C2f module, effectively tackling the complexities of multi-scale object detection. This integration has led to significant improvements in the model’s performance for road damage detection, demonstrating the effectiveness and efficiency of the proposed approach.

**Fig 4:**
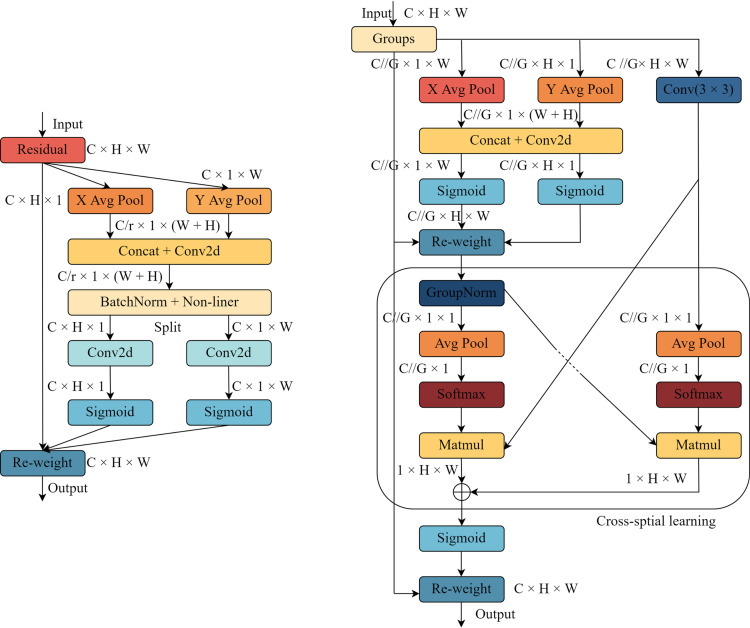
Schematic Diagram.

The Coordinate Attention (CA) attention module emerges as a formidable counterpart to the SE attention module, given that both mechanisms aspire to incorporate cross-channel information through the utilization of global average pooling operations. Fundamentally, global average pooling serves the purpose of compressing global spatial location information into channel descriptors, thereby generating channel-wise statistics. However, unlike the Squeeze-and-Excitation (SE) approach, the CA attention module distinguishes itself by incorporating spatial location information into the channel attention map, thereby enhancing the consolidation of features. Fig 4(a) offers a visual representation of the intricate architecture of the CA attention module.

Regarding the integration of coordinate information, the traditional global average pooling method, while compressing spatial data into channels, often neglects the significance of positional information. To address this limitation, a one-to-one dimensional feature encoding approach is employed. Consequently, the output of the *C*_*th*_ channel, possessing a height of *H*, can be reformulated into a more specific and informative form, thus preserving and utilizing critical positional details.


$$z_{C}^{H}(H)=\frac{1}{W}\sum_{0\leq i<W}x_{C}(H,i)$$ (7)

Similarly, the output of channel *C* with width *W* can be written as:


$$z_{C}^{W}(W)=\frac{1}{H}\sum_{0\leq j<H}x_{C}(j,W)$$ (8)

The integration of coordinate information and the subsequent formulation of coordinate attention effectively amalgamates features along varying spatial dimensions, leading to the creation of a direction-sensitive feature map. Once the coordinate attention information embedding is generated, the transformation outcomes are joined in a concatenated fashion. Subsequently, these features undergo a refinement process via the convolutional transform function F1, which is further enhanced by the application of the ReLU activation function, ultimately yielding a representation that is richer and more comprehensive:


$$f=\delta (F_{1}([z^{H},z^{W}]))$$ (9)

Decomposing f into 2 separate tensors along the spatial dimension and expanding their transformations yields the output of the CA:


$$y_{C}(i,j)=x_{C}(i,j)\times g_{C}^{H}(i)\times g_{C}^{W}(j)$$ (10)

where $g_{C}^{H}$ and $g_{C}^{W}$ are the attentional weights in the vertical and horizontal directions, respectively, and the two weights are added to the input feature map to enhance the representation of the feature map.

**Fig 5:**
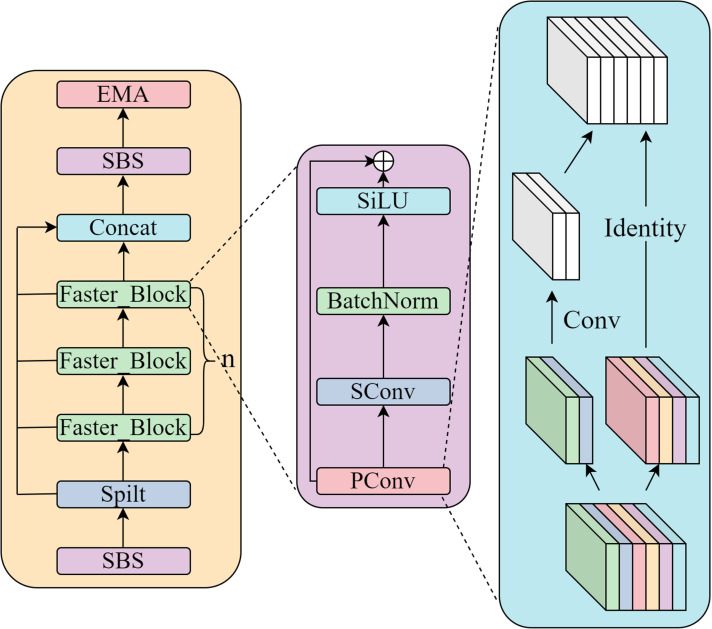
EMA_Faster_C2f Schematic Diagram.

Fig 4(b) offers a comprehensive visualization of the EMA attention module’s intricate structure. While the CA attention module impressively integrates spatial information into channel modeling, it fails to consider the crucial interplay between complete spatial locations. Furthermore, the limited receptive field of the 1 × 1 convolution hampers its ability to locally interact across channels and leverage contextual information. In contrast, the EMA module addresses these limitations by selecting the mutual elements of the 1 × 1 convolution within the CA module, referred to as the 1 × 1 branches. To effectively consolidate spatial structure information across multiple scales, the EMA module introduces a 3 × 3 kernel operating in parallel with the 1 × 1 branch, known as the 3 × 3 branch. This parallel sub-network structure enables the EMA module to preserve precise spatial structural information within each channel while capturing inter-channel information to regulate the significance of individual channels. Moreover, the EMA module employs a novel method of aggregating interspatial information using various directions across spatial dimensions, significantly enhancing feature aggregation. This process involves introducing two tensors: one for the output of the 1 × 1 dimension branch and another for the 3 × 3 dimension branch. To capture the holistic spatial knowledge within the outputs of the 1 × 1 branch, a 2D global mean pooling operation is utilized. Prior to the collaborative activation mechanism that incorporates channel characteristics, the output of the smallest branch undergoes a direct reshaping process to align with the desired dimensional structure. This approach ensures that the EMA module can comprehensively leverage spatial and channel information, resulting in a more robust and effective attention mechanism.To address the challenge of sluggish performance in the C2f module, we introduce the EMA_Faster_C2f module, which incorporates the proposed Faster Block in lieu of the traditional Bottleneck. The pConv operation within this block not only alleviates the computational load but also extracts features more efficiently compared to conventional convolutional methods, making it ideally suited for processing multi-channel features. This optimization notably reduces the model’s parameters and enhances its operational speed. As depicted in Fig 5, upon the feature map’s entry into the module, we initially employ the SBS module to adjust the number of channels in the feature map to align with the model’s requirements, thereby minimizing the number of parameters. Subsequently, we apply a split operation to divide the feature map, introducing additional branches and cross-layer connections. This augmentation enhances the gradient flow within the model, facilitating information propagation and enhancing model performance. After splitting, we utilize the Faster Block to perform enhanced feature extraction on a subset of the feature map. The Faster Block is primarily composed of operations such as PConv, SConv, and normalization. Specifically, within the Faster Block, we first employ PConv convolution for channel-specific feature extraction. Notably, we utilize traditional convolution to extract spatial features only on one-fourth of the input channels, maintaining the remaining channels unchanged. To ensure optimal utilization of information across all channels, we incorporate SConv after PConv. This layer facilitates the flow of feature information across all channels, preventing the model from losing crucial information while minimizing redundant computations. For subsequent operations, we opt for batch normalization as it provides faster inference compared to alternative methods. Furthermore, for the activation layer, we empirically select the SiLU activation function, commonly used in YOLO, to ensure compatibility with the overall model architecture.

### 3.3. AE- FPN

**Fig 6:**
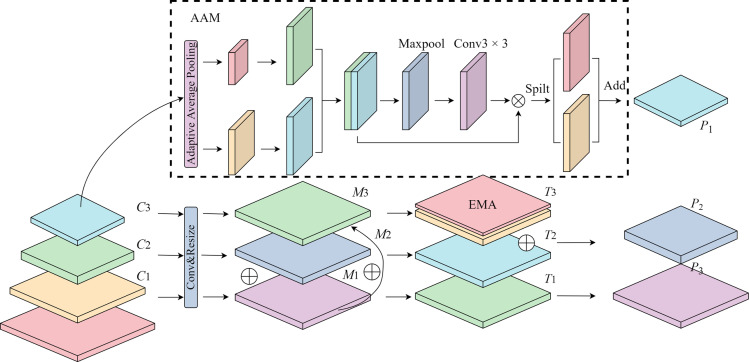
AE-FPN Schematic Diagram.

The original YOLO v8 model leverages the FPN-PAN architecture, an advanced variation of the conventional FPN structure. While the traditional FPN employs a top-down approach to propagate deep, high-level semantic information, it does not fully exploit the underlying location information. The FPN-PAN structure aims to address this limitation by incorporating additional operations. However, despite enriching semantic and location information, there are still opportunities for improvement. Firstly, the FPN-PAN structure lacks the ability to prioritize and focus on valuable information, which can lead to a decline in the quality of road defect detection. Additionally, during downsampling, the feature map tends to lose some original details, resulting in a relatively low reuse rate of information. To address these issues more effectively, this research proposes the Adaptive EMA Feature Pyramid Networks (AE-FPN). As illustrated in Fig 6, the AE-FPN structure aims to enhance the detection performance by adaptively emphasizing crucial features and maximizing the utilization of information across different scales.

**Fig 7:**
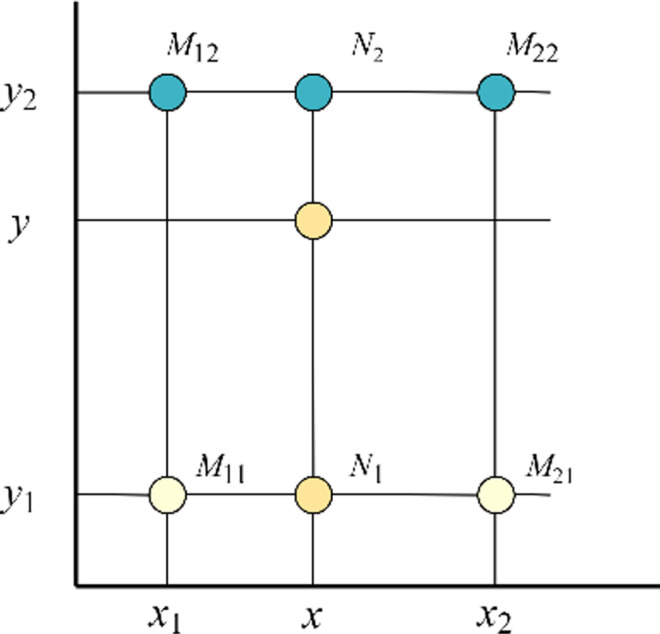
Schematic diagram of the bilinear interpolation algorithm.

Firstly, the three feature maps $\left[\begin{array}{ccc} {}*20cC_{1} & C_{2} & C_{3} \end{array}\right]$ are convolved 1 × 1 to unify the number of channels to 256. Secondly, $\left[\begin{array}{cc} {}*20cC_{2} & C_{3} \end{array}\right]$ feature layer is up-sampled by a bilinear interpolation algorithm, so that the $\left[\begin{array}{cc} {}*20cC_{2} & C_{3} \end{array}\right]$ feature layer has the same resolution as the $\left[C_{3}\right]$ feature layer, and the output feature layer is $\left[\begin{array}{ccc} {}*20cM_{1} & M_{2} & M_{3} \end{array}\right]$; The bilinear interpolation algorithm is a linear interpolation in two directions and the schematic principle is shown in Fig 7.

From the above Fig., let the value of the characteristic map at *P=(x,y)* be *f(P)*, Assume that the coordinates of the point to be interpolated are known to be (*x*,*y*), The four eigencenters as a function of the value of *M*_11_=(*x*_1_,*y*_1_)、*M*_12_=(*x*_1_,*y*_2_)、*M*_21_=(*x*_2_,*y*_1_)、*M*_22_=(*x*_2_,*y*_2_), are denoted as *f*(*M*_11_)、*f*(*M*_12_)、*f*(*M*_21_)、*f*(*M*_22_). First, linear interpolation in the *x*-direction is performed by making a straight line parallel to the *y*-axis over *P*, which intersects the straight *y* = *y*_1_ and with the *y* = *y*_2_ and at the points *N*_1_ and *N*_2_, respectively, to obtain the one-time linear estimates of the function f on and, *a* and *b*, where the formula for the calculation of *f*(*N*_1_) is shown in Eq.(11).


$$f(N_{1})=\frac{x_{2}-x}{x_{2}-x_{1}}f(M_{11})+\frac{x-x_{1}}{x_{2}-x_{1}}f(M_{21})$$ (11)

The same reasoning leads to *f(N*_*2*_*)*, The formula is shown in Eq(12).


$$f(N_{2})=\frac{x_{2}-x}{x_{2}-x_{1}}f(M_{12})+\frac{x-x_{1}}{x_{2}-x_{1}}f(M_{22})$$ (12)

Similarly, linear interpolation is performed in the y-direction to obtain the final result of bilinear interpolation, i.e., the bilinear estimate of the function *f* at the point *P*. The formula is shown in Eq.


$$f(P)=\frac{y_{2}-y}{y_{2}-y_{1}}f(N_{1})+\frac{y-y_{1}}{y_{2}-y_{1}}f(N_{2})$$ (13)

Then, the [*M*_1_] feature layer is added to the [*M*_2_] and [*M*_3_] feature layers by cross-layer fusion, and after the EMA module for important feature attention, the [*T*_1_ *T*_2_ *T*_3_] feature layer is output, the [*T*_2_] and [*T*_3_] feature layers are added to form the final [*P*_2_] prediction layer, and the [*T*_1_] feature layer is used as the [*P*_3_] prediction layer. Finally, the [*C*_3_] feature layer is fed into the AAM (Adaptive Attention Module) to get the [*P*_1_] prediction layer.AE-FPN strengthens the ability to focus on defects by introducing the EMA module and the AAM module and replaces the downsampling fusion method with cross-layer fusion, so that the feature maps retain the original feature information.The above two improvements improve the network’s ability to focus on road damage detection capability of small cracks and potholes.

### 3.4. LSC-Detect

**Fig 8:**
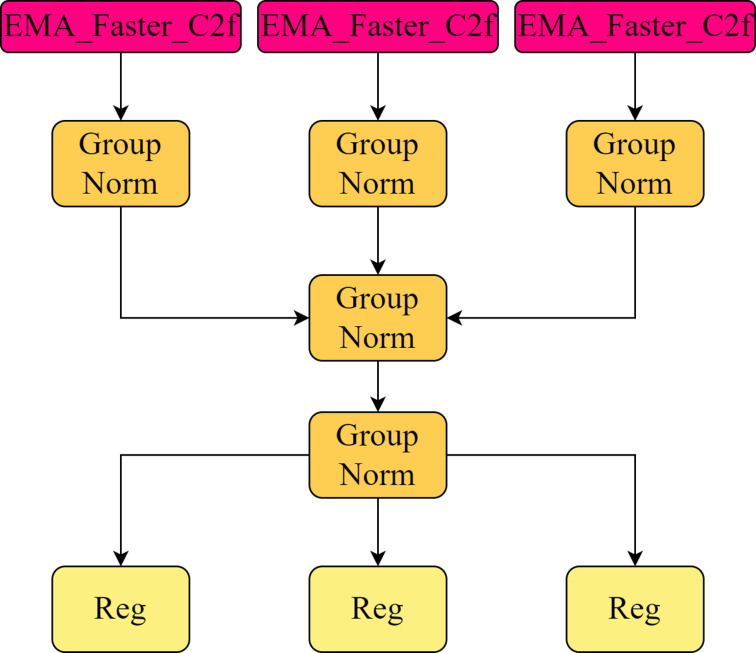
LSCD-Head module.

The original detection head design of YOLOv8 had some limitations. Specifically, the number of parameters in the detection head is relatively high, accounting for about one-fifth of the overall network computation. Each detection head needs to go through two 3 × 3 convolution and one 1 × 1 convolution to extract features, and this architecture significantly increases the number of model parameters. In addition, due to the traditional single-scale prediction method, this method is difficult to effectively deal with target detection tasks at different scales, because it only predicts at one feature level and fails to make full use of information from other scales. To solve these problems, a new Detection Head structure, Lightweight Shared Convolutional Detection Head (LSCD-Head), is proposed in this paper. The new structure uses GroupNorm convolution, a technique that has been validated in a FOCS paper, to enhance detection and classification. As shown in Fig 8, the key improvement to LSCD-Head is the use of a shared GroupNorm convolution instead of two separate layers of common convolution in each detection head. In addition, in order to solve the problem of the difference in the scale of the objects detected by different detection heads, a scale adjustment layer is added to deal with the features. Through these improvements, the new structure not only reduces the number of parameters, but also enhances the sensing ability of the detection head to multi-scale targets, making it more suitable for running on devices with limited computing resources.

## 4. Experimental results and analysis

### 4.1. Dataset

**Fig 9:**
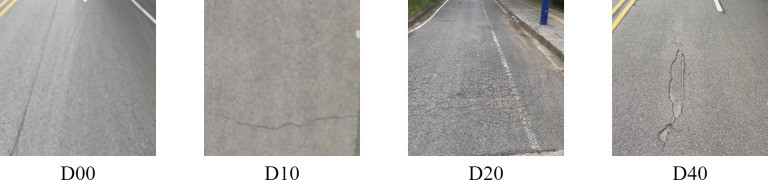
Sample plot of the dataset.

In this research, we use the RDD2022 dataset from the open-source Crowdsensing based Road Damage Detection Challenge (CRDDC 2022) for model training(Data | 2022 IEEE International Conference on Big Data) [33]. The RDD2022 dataset consists of 47,420 road images from six countries, namely, Japan, India, the Czech Republic, Norway, the United States, and China. The RDD2022 dataset consists of 47,420 road images from six countries: Japan, India, Czech Republic, Norway, USA and China. The dataset was prepared in the format of the PASCAL Visual Object Class (VOC) dataset, where XML files provide annotations for more than 55,000 road damage instances. As shown in Fig 9, this dataset contains four damage types: longitudinal cracks (D00), transverse cracks (D10), alligator cracks (D20), and potholes (D40). The distribution of these damage types suggests that longitudinal cracks are the most prevalent, while potholes are relatively less frequent. We divided the data from the RDD2022 training set into our experimental training set and validation set in a 7:3 ratio, using the RDD2022 test set as our experimental test set.

### 4.2. Evaluation metrics

**Fig 10:**
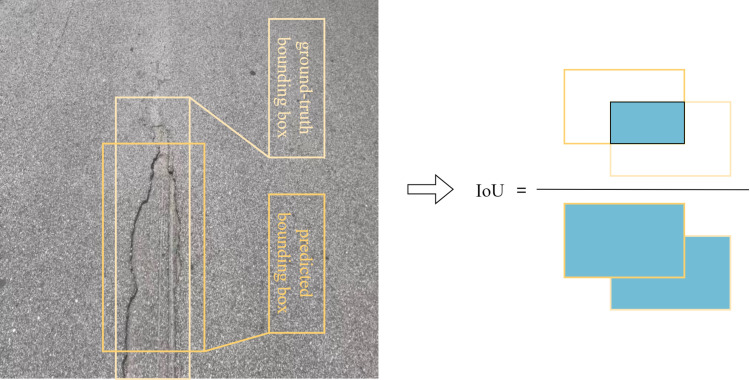
IoU Schematic.

In this research, we adhere to the established evaluation metrics for road damage detection outlined in the CRDDC2022 challenge. Specifically, we employ the mean average precision (mAP) as the primary criterion to gauge the performance of our trained model. Both precision and recall are inherently linked to the Intersection over Union (IoU) metric, which captures the degree of overlap between a predicted bounding box and the corresponding ground truth bounding box. The IoU is defined as the ratio of the intersection area between the predicted and true bounding boxes to their combined union area. This metric serves as an indispensible tool to quantify the alignment between the predicted target box and the actual target box, as illustrated in Fig 10 [34].

The mAP is the average of the average precision (AP) of different classes. In this research, mAP_50_ is used for evaluation. mAP_50_ represents the average accuracy at IoU threshold 0.5. It provides a comprehensive evaluation of the accuracy of the model in predicting the target box at different IoU thresholds. The specific parameters are as follows.

Precision (P): the precision is used to describe whether there is any misclassification in the detected positive samples, and is calculated as shown in Eq.


$$P=\frac{TP}{TP+FP}$$ (14)

Recall (Recall, R): The recall rate, also known as the check rate, is used to describe the model’s ability to recall positive samples, and is calculated as shown in Eq.


$$R=\frac{TP}{TP+FN}$$ (15)

### 4.3. IoU

During the training phase of defect detection, the locations of defects are precisely annotated by drawing rectangular bounding boxes. However, due to the inevitable variations in the positioning, there often exists an incomplete overlap between the candidate box and the manually labeled ground truth box. The objective of the training process is to refine the candidate box by adjusting the regression parameters outputted from the fully-connected layer, ensuring that it aligns closely with the true location of the defect. This iterative optimization process aims to generate increasingly accurate predictions at the conclusion of the training cycle. In the prediction stage, a similar challenge arises when locating steel surface defects, where the predicted bounding box may not perfectly overlap with the ground truth box. To quantify this degree of overlap and assess the performance of our model, we employ the Intersection over Union (IoU) metric. The IoU provides a numerical representation of the overlap between the predicted and true bounding boxes, and its calculation formula is presented in the following equation.


$$IoU=\frac{area(C)⋂area(GT)}{area(C)⋃area(GT)}$$ (16)

IoU represents the ratio of the intersection area between the predicted bounding box and the ground truth box to their combined area. As the IoU value increases, so does the accuracy of the prediction, indicating better performance. Commonly, when the IoU between the predicted and ground truth boxes surpasses a predefined threshold (for instance, IoU ≥ 0.5), it is deemed that the target has been successfully detected.

Parameters: The total number of learnable parameters in a neural network, encompassing model weights and biases, serves as a metric to gauge the spatial complexity and magnitude of the model. It provides insights into the model’s capacity to learn from the given data.

Giga-FLOPs (GFLOPs): This metric quantifies the number of billion floating-point operations executed by the model per second. It serves as an indicator of the computational complexity and efficiency of the model, offering valuable insights into its performance on different hardware platforms.

### 4.4. Experimental results

**Fig 11:**
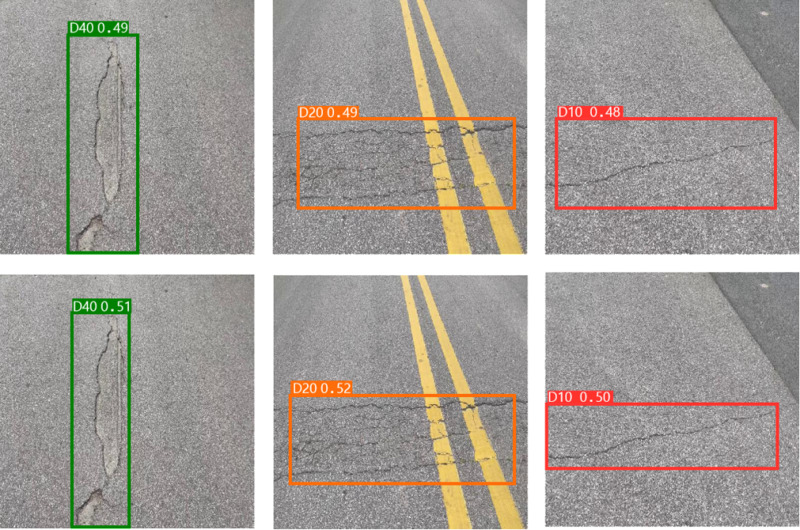
Ablation experiment.

To assess the efficacy of the individual modules introduced in our research, we conducted a series of ablation experiments. Initially, we constructed the SBS module as a substitution for the CBS module in the original network, naming the modified network YOLO v8l-SBS. This modified version served as the baseline for subsequent experiments. The results of these experimental data are shown in Table 1 and Fig 11, which provide insight into the performance gains achieved through the introduction of the SBS module.

**Table 1:** Ablation experiment.

Models	Params (M)	GFLOPs	Precision (%)	Recall (%)	mAP0.5 (%)
YOLO v8l	43.61M	164.8	58.2	53.2	56.4
YOLO v8l-SBS	23.27M	131.7	59.5	54.3	57.1

**Fig 12:**
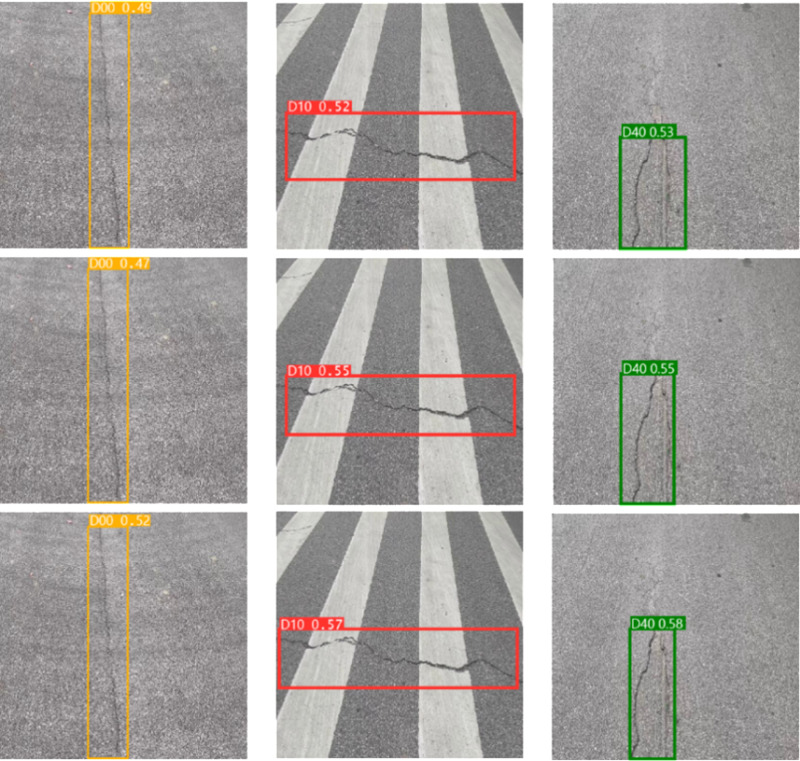
Ablation experiment.

In Fig 11, the bottom row represents the detection results of the YOLO v8l-SBS model, and the top row represents the detection results of the YOLO v8l model. As can be seen from Table 1 and Fig 11, replacing CBS module with SBS module greatly reduces Params and GFLOPs of the model, while improving mAP. In the evaluation indexes of Params and GFLOPs, the YOLO V8L-SBS model is 20.34M higher and 33.1 lower than the YOLO v8l model, respectively. In terms of mAP evaluation indexes, the YOLO v8l-SBS model was 0.7% higher than the YOLO v8l model. In D40, D20 and D10 defects, the YOLO v8l model with SBS module has a higher mAP than the original YOLO v8l model, and in particular, the accuracy and fit of the detection box is better than the original YOLO v8l model in the D10 defect. This remarkable achievement shows that we have succeeded in making the model lighter without compromising its detection accuracy. In addition, we focused on improving the Neck component of the model. For this purpose, the ablation experiments of EMA_Faster_C2f module and AE-FPN structure were carried out. The resulting models were named SBS-E, SBS-A, and SBS-EA, respectively. The results of these ablation studies are shown in Table 2 and Fig 12.

**Table 2:** Ablation experiment.

Models	Params (M)	GFLOPs	Precision (%)	Recall (%)	mAP0.5 (%)
SBS-E	20.17M	120.8	58.8	53.7	58.1
SBS-A	25.12M	137.9	59.7	55.2	59.3
SBS-EA	22.06M	127.2	61.5	57.6	61.3

In Fig 12, the top row represents the detection result of SBS-E, the middle row represents the detection result of SBS-A, and the bottom row represents the detection result of SBS-EA. As can be seen from Table 2 and Fig 12, by comparing the addition of EMA_Faster_C2f module and AE-FPN module, due to the introduction of attention mechanism in detecting D00 defects, the model can pay good attention to the feature information of shallow defects and improve the feature representation ability of the model. When detecting D10 and D40, thanks to the introduction of an adaptive pyramid structure, the model can better detect irregular and obvious defects based on the size and scale of the defects. In the detection of D10 defects, although both models are inferior to the SBS-EA model, it is obvious that the SBS-A model can detect the size of defects more accurately than the SBS-E model, and the accuracy of the detection frame is also higher, because AE-FPN can pay attention to the defect characteristics adaptively according to the size of defects. When detecting D40 defects, although the three models detect defects, the detection frame of SBS-A model is finer than that of SBS-E model, but all of them are slightly lower than SBS-EA model. Due to a large number of irregularities and obvious defects in the RDD2022 dataset, the SBS-E model with EMA_Faster_C2f module is 1.2% lower than the SBS-A model with AE-FPN module in terms of mAP evaluation indexes. While the introduction of the AE-FPN structure resulted in a slight increase in Params and GFLOPs, there was a marked improvement in mAP, which improved by 2.9% compared to the original model. Therefore, based on the above two test results, when the two modules are introduced at the same time, our model achieves a relatively uniform level in mAP, Params and GFLOPs, which not only ensures the ability to pay attention to shallow defects, but also improves the feature extraction ability of important semantic information of irregular and obvious defects. We conducted a comprehensive ablation experiment, and the experimental results are shown in Table 3.

**Table 3:** Stepwise ablation experiment.

Models	SBS	EMA_Faster_C2f	AE-FPN	LSCD-Head	Params (M)	mAP0.5 (%)
YOLO v8l					43.61	56.4
Case 1	√				23.27	57.1
Case 2	√	√			20.17	58.1
Case 3	√	√	√		22.06	61.3
Case 4	√	√	√	√	20.96	63.2

**Fig 13:**
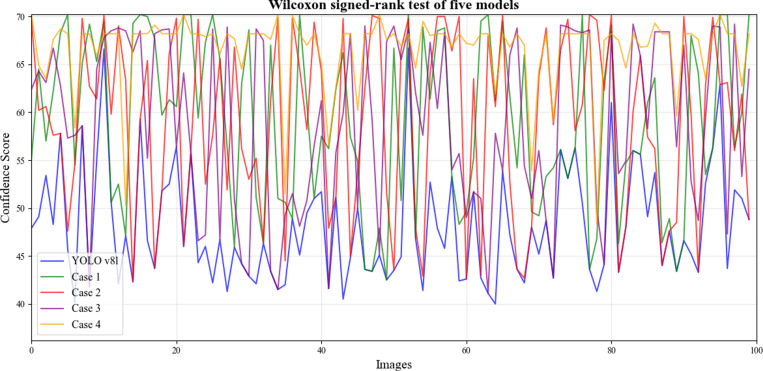
Wilcoxon Signed-Rank Test.

Cases 1–4 represent models derived from various combinations of module optimizations. SEA-YOLO v8 stands as the culmination of our efforts, incorporating all the designed modules. The overall performance of SEA-YOLO v8 exhibits significant improvements over the YOLO v8l baseline. As can be seen from Table 3, by adding SBS module, the number of model parameters is reduced by 20.34M, and mAP is increased by 0.7%, indicating that the proposed module greatly reduces the number of model parameters and makes the model more lightweight. The parameters of the model were reduced to some extent by adding LSCD-Head, and its mAP was increased by 1.9%, indicating that this module further improved the extraction ability of the model through the scale adjustment layer. To further verify the validity of each of our modules, we conducted a Wilcoxon Signed-Rank Test, and the experimental results are shown in Fig 13. First, we selected 100 images from the test data set, tested them on 5 models, and recorded the test results of each one. Then, we conducted a Wilcoxon Signed-Rank Test with the test results of Case1–4 and the baseline model yolov8l respectively. Finally, experimental results show that our proposed model is superior to the baseline model.

In order to further verify that our proposed model is lightweight, we conducted tests on different hardware platforms, and the experimental results are shown in Table 4. We tested FPS on three different hardware platforms: NVIDIA GeForce RTX 4060ti GPU, NVIDIA GeForce RTX 3060 GPU, and NVIDIA GeForce RTX 2080ti GPU. It can be seen from the experimental results that the SEA-YOLO v8 model proposed by us is better than the YOLO v8 model on three different hardware platforms. This experimental result further demonstrates that our proposed model is lighter than the baseline model yolov8l.

**Table 4:** Comparative experiment.

	Hardware platforms	FPS/Model
1	NVIDIA GeForce RTX 4060ti GPU	57.7/YOLO v8l
		82.1/Case 4
2	NVIDIA GeForce RTX 3060 GPU	39.4/YOLO v8l
		51.2/Case 4
3	NVIDIA GeForce RTX 2080ti GPU	48.2/YOLO v8l
		66.9/Case 4

In order to fully evaluate the optimization effect of each component and evaluate the performance of our SEA-YOLO v8 in road injury detection, we selected several representative algorithms for comparison, including the baseline model and variants of YOLO v8, detailed experimental results are shown in Table 5. Additionally, we have conducted experiments on Faster R-CNN, SSD, and YOLO v7-tiny. Faster R-CNN, a classical two-stage detection algorithm, enjoys widespread usage due to its effectiveness. SSD, on the other hand, represents a recent class of classical single-stage object detection algorithms. YOLO v7, introduced in 2022, is widely recognized as a state-of-the-art object detection algorithm. YOLO v7-tiny, a lighter version of YOLO v7, offers a compromise between accuracy and efficiency. Lastly, YOLO v5l, an enhanced variant of YOLO v5s, boasts an expanded network depth and width. The comprehensive experiments and comparisons allow us to gain a deeper understanding of the strengths and limitations of SEA-YOLO v8 in the context of road damage detection.

**Table 5:** Comparative experiment.

Models	Params (M)	GFLOPs	Precision (%)	Recall (%)	mAP0.5 (%)
YOLO v8l	43.61M	164.8	58.2	53.2	56.4
Faster R-CNN	41.48M	94.7	62.7	54.1	58.6
SSD	26.81M	60.9	59.1	49.7	53.2
YOLOv 8s	11.14M	28.7	57.6	52.7	54.9
YOLOv 7-tiny	6.02M	13.2	55.7	48.6	50.2
YOLOv 5l	46.53M	109.6	56.8	52.3	55.1
SEA-YOLO v8	20.96M	115.8	63.9	58.2	63.2

According to Table 4 of the experimental results, it can be seen that the mAP achieved by the Faster R-CNN model is closer to that achieved by the SEA-YOLOv8 model proposed by us. However, in terms of Params evaluation index, the Faster RCNN model is twice that of the SEA-YOLOv8 model proposed by us, which fully indicates that the SBS module proposed by us plays a very important role. On the other hand, although the YOLOv7-tiny model provides a more lightweight solution with fewer parameters, it lags behind our model in mAP by about 13%, which fully indicates that our proposed EMA_Faster_C2f module and AE-FPN module play an important role. In the comprehensive evaluation, our SEA-YOLOv8 model stands out. It reduces the number of Params and GFLOPs while maintaining a high mAP, achieving a significant balance between lightweight and precision. This makes the SEA-YOLO v8 model a satisfactory solution for the task at hand.

**Fig 14:**
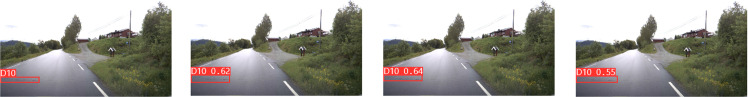
(a) ground truth, (b) YOLO v8l, (c) Faster R-CNN, (d) SSD.

**Fig 15:**
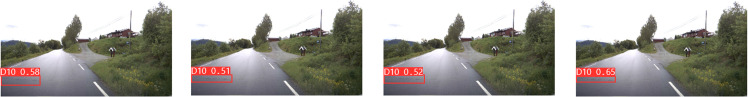
(a) YOLOv 8s, (b) YOLOv 7-tiny, (c) YOLOv 5l, and (d) SEA-YOLO v8.

**Fig 16:**
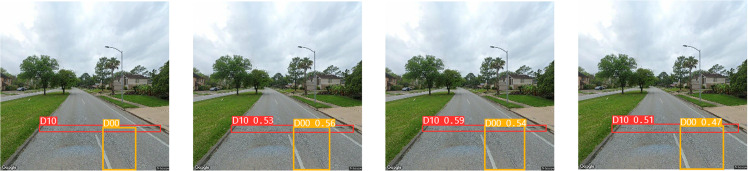
(a) ground truth, (b) YOLO v8l, (c) Faster R-CNN, (d) SSD.

**Fig 17:**
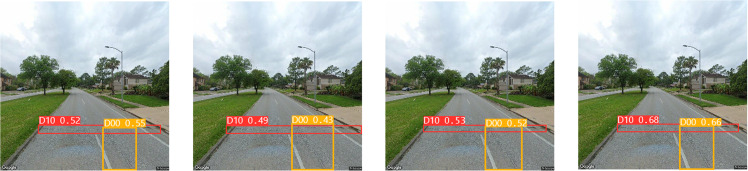
(a) YOLOv 8s, (b) YOLOv 7-tiny, (c) YOLOv 5l, and (d) SEA-YOLO v8.

**Fig 18:**
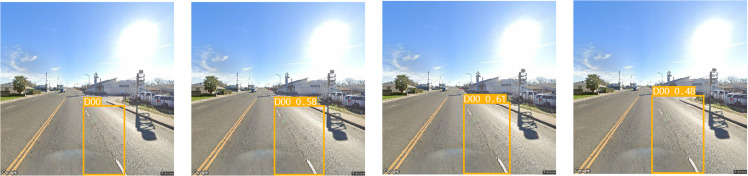
(a) ground truth, (b) YOLO v8l, (c) Faster R-CNN, (d) SSD.

**Fig 19:**
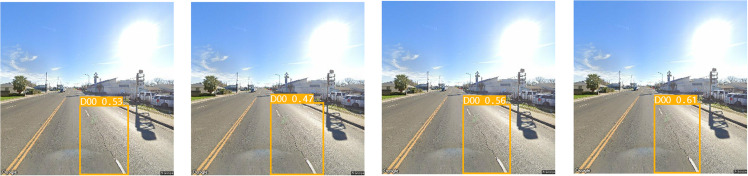
(a) YOLOv 8s, (b) YOLOv 7-tiny, (c) YOLOv 5l, and (d) SEA-YOLO v8.

**Fig 20:**
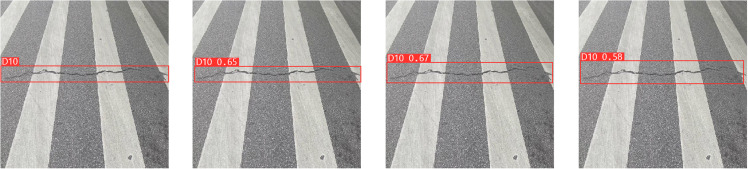
(a) ground truth, (b) YOLO v8l, (c) Faster R-CNN, (d) SSD.

**Fig 21:**
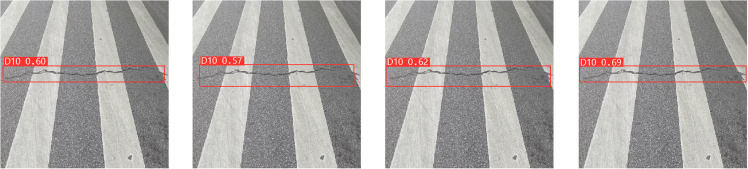
(a) YOLOv 8s, (b) YOLOv 7-tiny, (c) YOLOv 5l, and (d) SEA-YOLO v8.

Generally, the specifc conditions of roads are complex and varied, so the robustness of the model is particularly important. Algorithms that maintain good performance in complex road environments have better prospects for practical engineering applications. The detection results are shown in Figs 14–21. Correctly detecting long-distance crack damage is more diffcult than detecting ordinary crack damage. When all the algorithms have basically the same detection area, the accuracy of the SEA-YOLOv8 model proposed in this paper is more prominent. Although the model proposed in this paper is not as good as the two models YOLO v8s and YOLO v7-tiny in terms of lightweight, it is higher than them in terms of accuracy. Therefore, the models proposed in this paper balance each other in ensuring light-weighting without affecting the accuracy.

## 5. Discussion

To enhance the precision of road damage detection and minimize the labor and material costs associated with its assessment and maintenance, this research introduces an advanced road damage detection algorithm, designated as SEA-YOLO v8, which is an optimization of the YOLO v8 framework. This algorithm incorporates several crucial optimizations to bolster the model’s performance. Firstly, SBS modules are integrated into the backbone network and Neck structure, significantly reducing the model’s parameter count. This lightweighting approach enables the model to extract richer feature information related to road damage. Furthermore, the Neck structure is refined through the implementation of the EMA_Faster_C2f module. This innovation not only enhances the model’s efficiency but also maintains its ability to focus on critical defect features in road damage scenarios. Additionally, the AE-FPN structure is constructed to strengthen the model’s defect-targeting capabilities. By employing a cross-layer fusion method instead of the traditional down-sampling fusion, the feature map retains its original feature information, thereby improving the network’s proficiency in road damage detection. The LSC-Detect structure is used to enhance the detection capability of multi-scale targets. The experimental results demonstrate that SEA-YOLO v8 achieves a commendable mAP50 of 63.2% on the RDD2022 dataset. This performance not only surpasses other algorithms in terms of detection accuracy, but it also boasts a more concise parameter set.

### 5.1. Research limitation

However, despite the remarkable performance of SEA-YOLO v8, there are still areas for improvement. Given the extensive diversity of road damage types in real-world scenarios, the RDD2022 dataset, which only covers four common road damage types, may limit the algorithm’s generalizability. Future work could explore the inclusion of a more comprehensive range of road damage types to further enhance the algorithm’s robustness and accuracy. In the current study, our model, SEA-YOLO v8, is limited to recognizing four specific road damage types due to the constraints of the RDD2022 dataset. While we have made efforts to reduce the model’s parameter count through the introduction of SBS modules and the EMA_Faster_C2f module, we recognize that YOLO v8l, chosen as our baseline, remains relatively large and may pose challenges for deployment on resource-constrained mobile endpoint devices that require high real-time performance. Looking ahead, we aim to expand our dataset to incorporate a more diverse range of road damage types, thus enhancing the model’s generalizability and applicability. Furthermore, we will continue to optimize the network model to minimize its size and complexity, while maintaining or even improving detection speed and accuracy. Our ultimate goal is to develop a robust and efficient model that can be effectively used for practical applications, such as pavement crack and pothole detection, to support road maintenance efforts and improve safety standards, and to make a greater contribution to sustainable urban development.

## 6. Conclusion

Based on YOLO v8 network, a SEA-YOLO v8 model is proposed in this paper. The SBS module and EMA_Faster_C2 module are introduced in this model, which not only greatly reduces the parameters of the model, but also captures valuable significant features of the road defect area, and effectively inhibits information unrelated to road defects. In addition, the construction of AE-FPN structure and LSC-Detect structure not only enhances the sensing ability of the detection head to multi-scale targets, but also enhances the defect location ability of the model. The experimental results show that:

(1) The improved YOLO v8 detection model shows excellent performance, which is higher than the mainstream target detection algorithms such as YOLO v7-tiny and YOLO v5 in terms of mAP50, Precision and Recall evaluation indexes.
(2) The introduction of SBS module and EMA_Faster_C2 module not only greatly reduced the parameters of the model, but also effectively suppressed the information unrelated to road defects.
(3) This method can effectively detect road defects, accurately detect D00, D10, D20 and D40 road defects, and the detection accuracy is high.

## Supporting information

S1 Data Data. (Zip)
